# Allogeneic hematopoietic cell transplantation is curative in CARMIL2 deficiency

**DOI:** 10.70962/jhi.20250200

**Published:** 2026-07-29

**Authors:** Johannes Raedler, Florian Gothe, Thomas Magg, Philipp Peters, Karen Helene Bronken Martinsen, Ursula Holzer, Ayca Kiykim, Ayse Kalyoncu Ucar, Roshini S. Abraham, Hemalatha G. Rangarajan, Adeeb NaserEddin, Polina Stepensky, Irina Zaidman, Ehud Even-Or, Joern-Sven Kuehl, Daniel Graefe, Venetia Bigley, Christo Tsilifis, Anders Fasth, Nizar Mahlaoui, Oliver Wegehaupt, Carsten Speckmann, Jean-Laurent Casanova, Christoph Klein, Bénédicte Neven, Vivien Béziat, Romain Lévy, Fabian Hauck

**Affiliations:** 1 Department of Pediatrics, Dr. von Hauner Children’s Hospital, LMU University Hospital, LMU Medizin, LMU Munich, Munich, Germany; 2 Department of Pediatric and Adolescent Medicine, Oslo University Hospital Rikshospitalet, Oslo, Norway; 3 Children’s Hospital, University of Tübingen, Tübingen, Germany; 4 Pediatric Immunology and Allergy, Istanbul University-Cerrahpasa, Cerrahpasa School of Medicine, Istanbul, Turkey; 5 Radiology, Cerrahpasa School of Medicine, Istanbul University-Cerrahpasa, Istanbul, Turkey; 6 Deptartment of Pathology and Laboratory Medicine, Nationwide Children’s Hospital, Columbus, OH, USA; 7 Division of Hematology, Oncology and Bone Marrow Transplant, Department of Pediatrics, Nationwide Children’s Hospital, Columbus, OH, USA; 8 Department of Bone Marrow Transplantation, Faculty of Medicine, Hadassah Medical Center, Hebrew University, Jerusalem, Israel; 9 Department of Women and Child Health, Hospital for Children and Adolescents, Leipzig University Hospital, Leipzig, Germany; 10 Institut for Pediatric Radiology, Leipzig University Hospital, Leipzig, Germany; 11 Translational and Clinical Research Institute and NIHR Newcastle Biomedical Research Centre, Newcastle University and Newcastle upon Tyne Hospitals NHS Foundation Trust, Newcastle upon Tyne, UK; 12 Paediatric Hematopoietic Stem Cell Transplant Unit, Great North Children’s Hospital, Newcastle Hospitals NHS Foundation Trust, Newcastle upon Tyne, UK; 13 Translational and Clinical Research Institute, Newcastle University, Newcastle upon Tyne, UK; 14 Department of Pediatrics, Institute of Clinical Sciences, University of Gothenburg, Gothenburg, Sweden; 15 Department of Pediatric Rheumatology and Immunology, Queen Silvia Children’s Hospital, Gothenburg, Sweden; 16 French National Reference Center for Primary Immune Deficiencies, Necker Enfants Malades University Hospital, Assistance Publique-Hôpitaux de Paris, Paris, France; 17 Pediatric Immunology-Hematology and Rheumatology Unit, Assistance Publique-Hôpitaux de Paris, Hôpital Necker-Enfants Malades, Paris, France; 18 Faculty of Medicine, Institute for Immunodeficiency, Center for Chronic Immunodeficiency, Medical Center, University of Freiburg, Freiburg, Germany; 19 Department of Pediatric Hematology, Oncology and Stem Cell Transplantation, Faculty of Medicine, Children’s Hospital, Medical Center, University of Freiburg, Freiburg, Germany; 20 St. Giles Laboratory of Human Genetics of Infectious Diseases, Rockefeller Branch, The Rockefeller University, New York, NY, USA; 21 Université Paris Cité, Institut Imagine, Laboratory of Human Genetics of Infectious Diseases, Necker Branch, INSERM UMR 1163, Paris, France; 22 Service de Pédiatrie, Assistance Publique-Hôpitaux de Paris, Hôpital Necker-Enfants Malades, Paris, France; 23 Howard Hughes Medical Institute, New York, NY, USA; 24 German Center for Child and Adolescent Health, Munich, Germany; 25 University Children’s Hospital Mannheim, Ruprecht-Karls-Universität Heidelberg, Heidelberg, Germany

## Abstract

Biallelic loss-of-function variants in capping protein regulator and myosin 1 linker 2 (*CARMIL2*) cause a complex disorder of immune dysregulation hallmarked by susceptibility to infections, inflammatory bowel and skin disease, and Epstein-Barr virus–positive smooth muscle tumors (EBV+ SMTs). We report a multicenter retrospective study to evaluate hematopoietic cell transplantation (HCT) outcomes in CARMIL2-deficient patients. 17 patients underwent 19 HCTs, with a total follow-up of 768 mo and a median follow-up of 37 mo (range 1–195). Three patients died during the early posttransplant period (overall survival, 82.4%), and two required a second HCT for graft failure. Despite limitations due to cohort size and high pre-transplant morbidity in individual patients, HCT improved all major disease manifestations, including infections, inflammatory disease, and previously treatment-refractory EBV+ SMTs. No patient required immunoglobulin replacement after HCT, and no detrimental effects of mixed chimerism were noted. Allogeneic HCT is therefore a curative option for patients with CARMIL2 deficiency, which should be offered upon diagnosis.

## Teaser

Allogeneic HCT substantially addresses immunodeficiency, immune dysregulation, and Epstein-Barr virus–positive smooth muscle tumors in patients with CARMIL2 deficiency and should be offered shortly following diagnosis.

## Graphical Abstract

**Figure:**
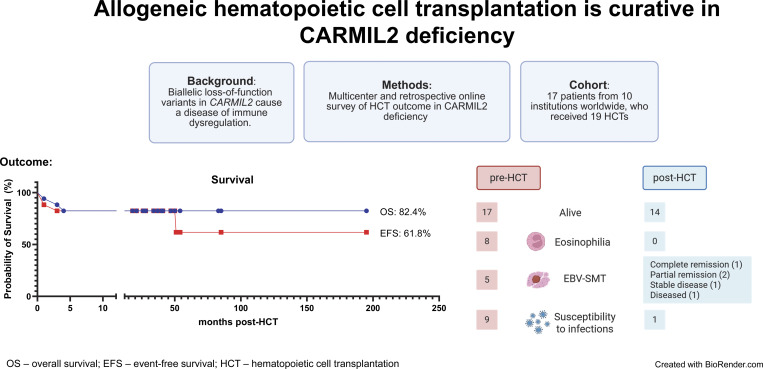

## Introduction

Capping protein regulator and myosin 1 linker 2 (CARMIL2), previously known as RGD, leucine-rich repeat, tropomodulin, and proline-rich–containing protein (RLTPR) (1), is a multidomain cytosolic protein. Biallelic loss-of-function (LOF) variants in human *CARMIL2* cause a polymorphic combined immunodeficiency (CID) (2, 3, 4, 5). To date, an estimated 120 patients have been reported in the literature (2, 3, 4, 5, 6, 7, 8, 9, 10, 11, 12, 13, 14, 15). The hallmarks of the disease include susceptibility to infections, including mycobacteria, and immune dysregulation with inflammatory bowel disease (IBD) and dermatitis, failure to thrive, and a predisposition to Epstein-Barr virus–positive smooth muscle tumors (EBV+ SMTs) (5, 7, 9, 16). Immunological studies reveal decreased memory B, natural killer (NK), and central memory CD4^+^ and CD8^+^ T cells, low levels of regulatory T cells (Tregs), and low or absent T cell proliferation after stimulation with CD3/CD28 (2, 4, 5, 17). Based on the mouse model (18), human CARMIL2 deficiency was initially believed to be caused by deficient CD28-mediated T cell co-simulation (17). This was underlined by successful rescue of T cell proliferation following supplementation of interleukin-2 to CARMIL2-deficient human cells (5, 19). However, recent data support a broader clinical and immunological phenotype of human CARMIL2 deficiency compared to CD28 deficiency alone (5, 20). Although the complete underlying mechanisms of CARMIL2 deficiency remain unsolved, it has been postulated that CARMIL2 controls NF-kB activation downstream of protein kinase C–dependent receptors in T cells and beyond (5).

The high rates of mortality (60% survival at the age of 40 years) and severity of infectious and inflammatory comorbidities observed under conservative treatment emphasize the need for definitive treatment options for CARMIL2 deficiency (4, 5). Allogeneic hematopoietic cell transplantation (HCT) is recognized as the gold standard treatment for a variety of severe pediatric inborn error of immunity (IEI) (21, 22), but its benefit in patients with CARMIL2 deficiency remains unclear. Successful HCT for CARMIL2 deficiency has been reported in a single case by Rastogi et al. (8), whereas an unsuccessful HCT was reported by Yonkof et al. (9). To our knowledge, no systematic review of HCT for CARMIL2 deficiency has been reported to date. We present a multicenter and retrospective analysis of HCT outcomes for 17 patients with confirmed CARMIL2 deficiency.

## Results

### The international CARMIL2 deficiency HCT cohort

We report on HCT in 17 patients (9 male, 8 female) from 11 kindreds and 10 institutions as detailed in Table 1. Biallelic *CARMIL2* LOF variants were identified by next-generation sequencing with verification by Sanger sequencing (5). Patient 4 (P4), who carried a compound heterozygous missense variant and frameshift-causing indel, showed revertant CD4^+^ T cells due to reading frame restoration by an additional nucleotide insertion at the indel site (5). Heterozygous somatic reversion to the wild-type allele was documented in P14 (5). All patients, except for P17, were previously reported by Lévy et al. (5). P1 has also been reported by Yonkof et al. (9). P3 was diagnosed postmortem, leading to identification of affected siblings (P15 and P16). P17 was diagnosed after HCT.

**Table 1.** Patient and HCT characteristics

Patient	Gender	*CARMIL2* variants (consequences)	Outcome	Follow-up (months after HCT)	Age at HCT (months)	Donor	HLA-match	Stem cell source	Stem cell dose (CD34^+^ ×10^-6^/kg BW)	Conditioning regimen	Sero-therapy	Engraftment (days after HCT)			GVHD prophylaxis	aGVHD	Complications
												WBC	NEU	PLT			
1	Male	Homozygous c.1256_1285del (p.Q419_L428del)	Deceased (respiratory failure)	1	144	MUD	10/10	PBSC (TCR αβ and CD19 depleted)	5.5	Bu Flu TT	ATG RTX	12	12	–	None	No	AKI (grade 3), engraftment syndrome, multifactorial respiratory failure: progression EBV-SMTs, PjP, CMV, candida
2	Male	c.1825G>A (p.D609N); c.249+1G>T (splicing)	Deceased (JC virus encephalitis)	3	450	MUD	10/10	PBSC	NA	Bu Flu	Alem	–	14	26	CSA	No	JC virus encephalitis
3*	Male	Hhomozygous c.118_119insA (p.N41Kfs47*)	Deceased (ARDS)	4 (after 2nd HCT)	1st: 27	mMRD (father)	5/10	BM	7.1	Bu (23478) Flu 160	Alem RTX	Primary graft failure			PTCY CSA MMF	No	*Staphylococcus aureus* bacteremia, CMV, ADV, BKV
					2nd: 28	MRD (mother)	10/10	BM	8.54	Flu 160	Alem	-	-	-	CSA MMF	No	ARDS (d +7)
4	Male	c.887_897delins TGTTGTCCTG (p.S296Mfs*10); c.1874T>C (p.L625P)	Alive	18	215	MRD (sibling; *WT/UN*)	10/10	BM	19.0	Treo 42 Flu 160 TT 8	ATG	21	28	25	CSA MTX	No	EBV, ADV
5^$^	Male	Homozygous c.1149+5G>C (splicing)	Alive	21	68	MUD	10/10	PBSC	11.6	Treo 42 Flu 150	ATG RTX	23	13	18	CSA MTX	Grade I (S1, L0, G0)	CMV
6^%^	Male	Homozygous c.691_715del GCCTTGAGGTCTCAGAACAGATTCT (p.L231Tfs*2)	Alive	26	118	MUD	10/10	BM	2.8	Treo Flu TT	ATG	21	21	58	CSA MTX	Grade I (S2, L0, G0)	AKI, CMV
7^$^	Male	Homozygous c.1149+5G>C (splicing)	Alive	28	110	MUD	10/10	PBSC	8.4	Treo 42 Flu 150	ATG RTX	21	11	17	CSA MTX	Grade I (S1, L0, G0)	None
8	Female	c.926T>C (p.L309P); c.1071+2T>A (Splicing)	Alive	32 (after 2nd HCT); overall: 83	1st: 384	MUD	10/10	PBSC	6.1	Mel 140 Flu 150	Alem	11	11	12	CSA	No	Secondary graft failure, Idiopathic neuropsychiatric episode (7 mo), FUO, EBV
					2nd: 435 (51 after 1st)	MUD (same donor)	10/10	PBSC	6.1	Treo 42 Flu 150 TT 10	Alem	11	11	11	CSA MMF	No	Idiopathic psychotic episode, pulmonary MAC, oral candidosis
9^#^	Female	Homozygous c.958+1G>C (splicing)	Alive	34	85	MRD (sibling; *WT/WT*)	10/10	BM	5.9	Treo 42 Flu 150 TT 5	ATLG	22	24	24	CSA MMF	Grade I (S1, L0, G0)	Lung and GI disease, CMV
10^§^	Male	Homozygous c.871G>C (splicing)	Alive	37	42	MRD (sibling; *UN/UN*)	10/10	BM	5.6	Treo 42 Flu 150	None	15	19	23	CSA MTX	Grade II (S3, L0, G0)	*S. aureus* bacteremia, CMV, ADV, MPV
11^§^	Female	Homozygous c.871G>C (splicing)	Alive	40	108	MUD	9/10	BM	7.3	Treo 42 Flu 150	ATG	21	21	NA	CSA MTX	Grade II (S3, L0, G0)	CMV, HSV, HHV6 (viremia, spinal) with limbic encephalitis, epilepsy, poor graft function
12^%^	Female	Homozygous c.1149+5G>C (splicing)	Alive	41	99	MRD (sibling; *WT/MUT*)	10/10	BM	5.0	Bu Flu	ATG	16	13	22	CSA MMF	No	CMV, EBV
13^#^	Male	Homozygous c.958+1G>C (splicing)	Alive	47	193	MUD	9/10	PBSC	12.7	Treo 42 Flu 150 TT 10	None	11	16	78	PTCY TAC	Grade IV (S3, L4, G4)	CMV, EBV, engraftment syndrome
14	Female	Homozygous c.1856T>C (p.L619P)	Alive	50	201	MUD	10/10	PBSC (TCR αβ and CD19 depleted)	8.9	TBI 4 Gy Flu 150 TT 10	ATG	18	18	73	PTCY	No	CKD (5, chronic dialysis), PRES, FUO, CMV, HSV, BKV (multivirus-specific T cells)
15*	Female	Homozygous c.118_119insA (p.N41Kfs47*)	Alive	54	5	MRD (father)	10/10	BM	14.2	Bu (18826) Flu 160	ATG	16	12	41	CSA MMF	No	SOS, mucositis grade 4, FUO; EBV, enterovirus
16*	Female	Homozygous c.118_119insA (p.N41Kfs47*)	Alive	85	10	MRD (father)	10/10	BM	13.7	Bu (21848) Flu 160	ATG	27	24	36	CSA MMF	No	ARDS, *Klebsiella pneumoniae* bacteremia, CMV, EBV
17	Female	Homozygous c.1636del (p.C546Vfs*13)	Alive	195	26	mMRD (mother)	5/10	BM (CD3 depleted)	6.5	Bu Cy	ATG RTX	33	33	33	None	No	None

*CARMIL2* variants were annotated using the canonical human transcript of isoform 3 (NCBI Reference Sequence: NM_001438835.1) described earlier (5). All variants, except P17, have been reported in Table S2 by Levy, Gothe et al. (2022) (5). *CARMIL2* genotypes of healthy sibling donors are annotated as *WT/MUT* (heterozygous carrier), *WT/WT* (noncarrier), *WT/UN *(carrier status is unknown), or UN/UN (no genetic testing performed). Healthy parent donors are assumed to be heterozygous carriers of *CARMIL2* variants. aGVHD, acute GVDH (S, skin; L, liver; G, gastrointestinal); AKI, acute kidney injury; Alem, alemtuzumab (in mg/kg); ATG, antithymocyte globulin; ATLG anti-T lymphocyte globulin; BKV, BK virus; BU, busulfan (reported as AUC [area under the concentration]-time curve in µmol × min/L); BW, body weight; CSA, cyclosporine; Cy, cyclophosphamide (in mg/kg); Flu, fludarabine (in mg/m2); HLA, human leukocyte antigen; MEL, melphalan (in mg/m2); MMF, mycophenolate mofetil; mMRD, mismatched related donor; MTX, methotrexate; NA, not available; NEU, neutrophils; PLT, platelets; PRES, posterior reversible encephalopathy syndrome; PTCY, post-transplantation cyclophosphamide; RTX, rituximab; TAC, tacrolimus; TBI, total-body irradiation; Treo, treosulfan (in g/m2); TT, thiotepa; WBC, white blood cells; HHV6, human herpes virus type 6; HSV, herpes simplex virus; MAC, *M. avium* complex. “–” not reached; *, $, #, and § denote sibling; % denotes cousins. Engraftment was defined as WBC >1,000/μl, NEU >500/μl, and PLT >50,000/μl on 3 consecutive days.

### HCT characteristics

Indication for HCT was immunodeficiency in 16 (including infections or immune dysregulation) and preemptive in one (P15), following the genetic diagnosis of a deceased sibling P3. We report on a total follow-up of 768 patient months after HCT with a median of 37 mo (range 1–195). HCT characteristics are summarized in Table 1 and outlined below. Median age at HCT was 108 mo (range 5–450). Donors were seven HLA-matched unrelated donors (MUD, 10/10), six matched related donors (MRD, 10/10), two partial MUD (9/10), and two haploidentical parent donors (mMRD, 5/10). Healthy parent donors are assumed to be heterozygous carriers of *CARMIL2* variants, whereas *CARMIL2* genotypes of sibling donors are annotated in Table 1. We observed no benefit of preferring *CARMIL2* homozygous wild-type donors over healthy carriers of *CARMIL2* variants. Grafts originated from bone marrow (BM) in 10 and peripheral blood stem cells (PBSCs) in 7 HCTs. Graft manipulation was performed by TCRαβ and CD19 depletion (P1 and P14; PBSC from MUD) or CD3 depletion (P17; BM from 5/10 mMRD). Median CD34^+^ stem cell dose was 7.2 × 10^6^/kg body weight (range: 2.8–19.0).

As per European Society for Immunodeficiencies/European BM Transplantation (EBMT) Inborn Errors Working Party guidelines (23), conditioning regimens were treosulfan-based in eight (four myeloablative with thiotepa, four reduced intensity) and busulfan-based in seven (two myeloablative, one sub-myeloablative, four doses unknown). Other patients received melphalan or total-body irradiation (4 Gy). Three out of seven patients (42%) receiving busulfan-based conditioning died, accounting for all deaths in this cohort. Serotherapy was used in all patients except for P10 and P13 (11 antithymocyte globulin, 3 alemtuzumab, and 1 antilymphocyte globulin). Post-HCT cyclophosphamide was administered in three patients. Graft-versus-host disease (GVHD) prophylaxis was none (2) or calcineurin inhibition (cyclosporin or tacrolimus, 14) in combination with methotrexate (6) or mycophenolate mofetil (5).

### HCT outcome

**Figure 1.**
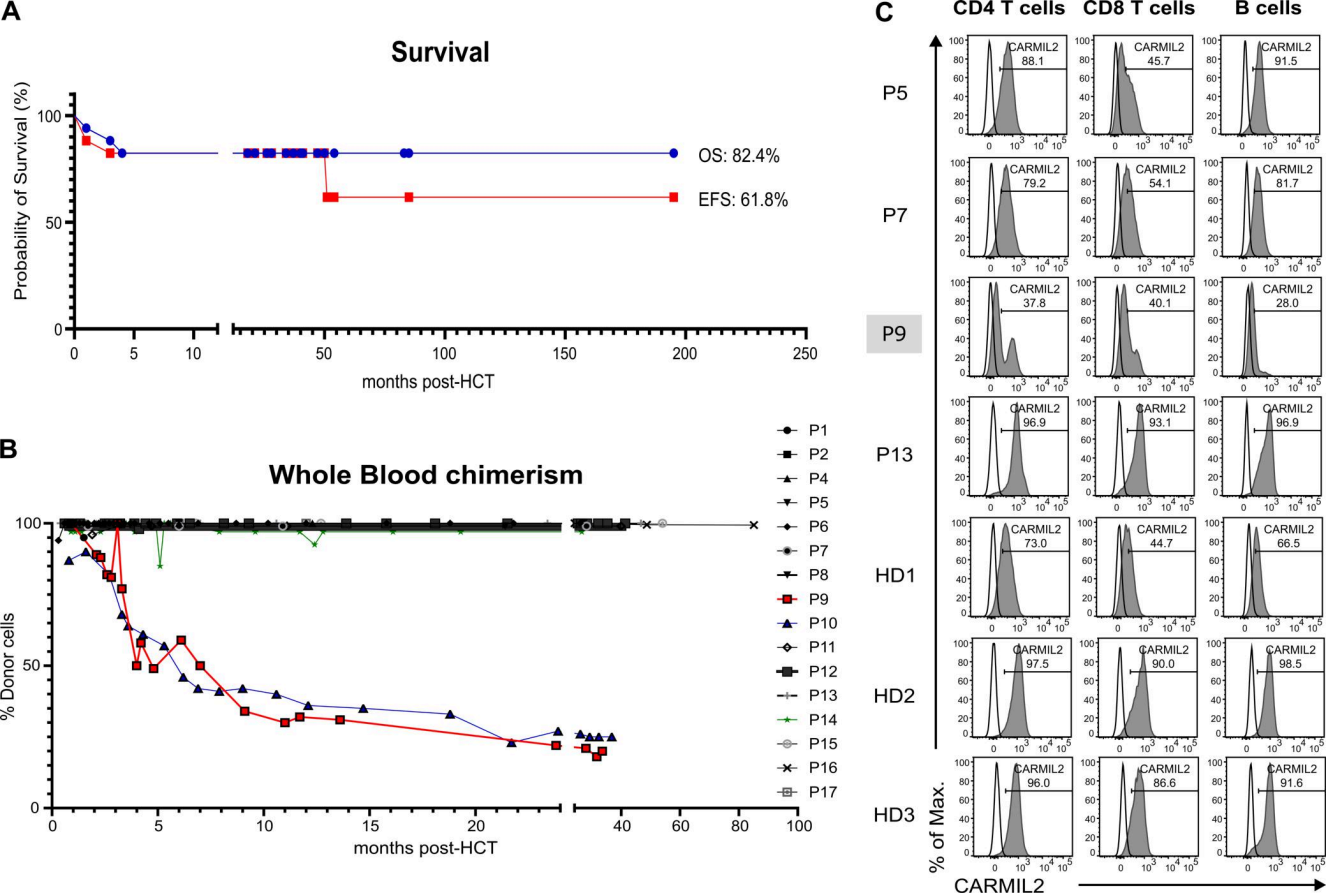
**HCT corrects CARMIL2 deficiency. (A)** Kaplan–Meier survival curve demonstrates long-term OS after HCT of 82.4% (14 of 17 patients) and EFS (events defined as death of any cause or graft failure) of 61.8% censored at last reported follow-up. **(B)** Whole blood donor chimerism results are depicted for each patient in months after HCT demonstrating stable full donor chimerism in the majority of surviving patients. Patients with mixed chimerism are emphasized in color. **(C)** Flow cytometry shows normalization of intracellular CARMIL2 expression for P5, P7, and P13, compared to respective healthy controls (HD). HD1 is a travel control for P5 and P7, and HD3 is the healthy control for P13. P9 with mixed donor chimerism, emphasized by a gray background, shows reduced intracellular CARMIL2 expression compared to HD2.

Three patients died within 4 mo after HCT due to multifactorial respiratory failure (P1), JC virus encephalitis (P2), and idiopathic acute respiratory distress syndrome (ARDS) (P3, following a second HCT), resulting in an overall survival (OS) of 82.4% (Fig. 1 A). HCTs for deceased patients and surviving patients P9–P17 was performed in a similar timeframe (Table S2). Overall event-free survival (EFS) was 61.8% (Fig. 1 A), with events defined as death of any cause or graft failure. We report on 19 HCTs as P3 (age 27 and 28 mo) and P8 (age 384 and 435 mo) required a second HCT for primary (P3) and secondary (P8) graft failure. Median age at first HCT did not differ significantly for deceased patients (144 mo, range 27–450) and surviving patients (103 mo, range 5–384; P = 0.5088). Median engraftment occurred on day +19.5 for leukocytes (range: 11–33), day +16 for neutrophils (range: 11–33), and day +25 for platelets (range: 11–78).

**Figure S1.**
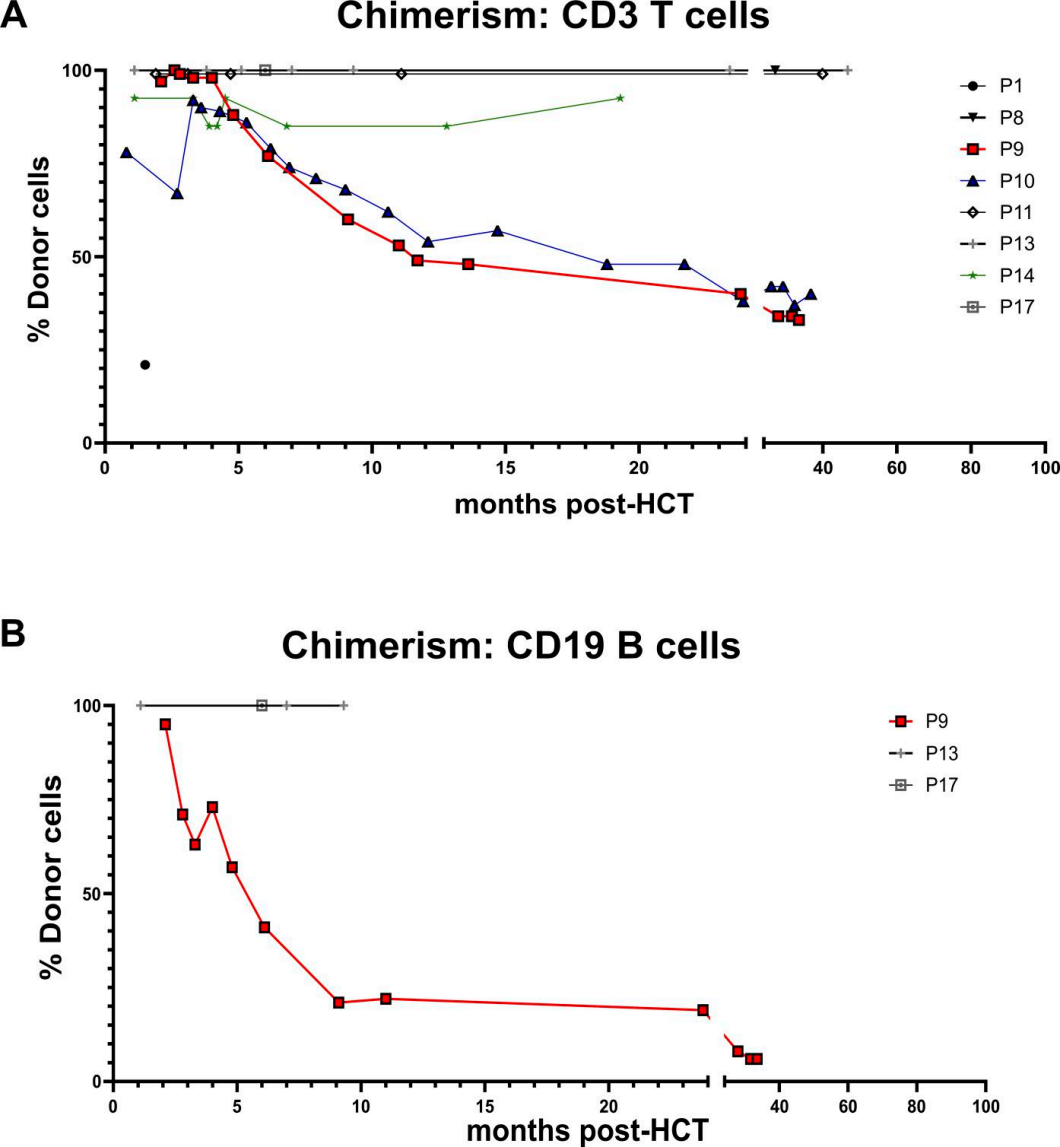
**Lineage-specific chimerism results. (A and B)** Donor chimerism results are depicted for CD3 T cells (A) and CD19 B cells (B) for each patient, when available, in months after HCT.

Full donor chimerism was achieved in all but two patients (Fig. 1 B; and Fig. S1, A and B, P9 red, P10 blue). P14 (Fig. 1 B, green) spontaneously corrected to full donor chimerism. Intracellular CARMIL2 staining was available in four patients after HCT (Fig. 1 C), three of which displayed normal CARMIL2 expression, corresponding to full donor chimerism. P9 exhibited bimodal CARMIL2 expression, particularly in CD4^+^ and CD8^+^ T cells, reflecting the mixed global and lineage-specific donor chimerism. Slightly reduced CARMIL2 expression in CD8^+^ T cells in P5 and P7 likely resulted from pre-analytic shipment and appeared normal compared to the corresponding healthy donor (HD1, Fig. 1 C). Mixed chimerism did not appear to be associated with a worse clinical outcome.

Acute GVHD (aGVHD) occurred in seven patients (41% overall aGVHD, 18% grade II–IV; 2/12% grade II, and 1/6% grade IV [24]). P13 developed severe aGVHD (grade IV: skin 3, liver 4, gut 4; PBSCs from a 9/10 MUD) requiring intensified immunosuppression until day +528. P13 also presented with persistent indirect hyperbilirubinemia; however, chronic GVHD has not been reported for any patient. P13 (MUD 9/10) and P10 (MRD 10/10) were the only two patients not receiving serotherapy during conditioning. Notably, all patients with aGVHD had received treosulfan-based conditioning.

Treatment-related infectious complications included reactivation or infection with cytomegalovirus (CMV; 11 total, 9 alive), EBV (7 total, 7 alive), and adenovirus (3 total, 2 alive). Additionally, P11 had human herpes virus type 6–related limbic encephalitis 2 mo after HCT, resulting in autonomic dysfunction, impaired short-term memory, and treatment-refractory epilepsy, requiring long-term anti-infective prophylaxis. P14 received multivirus-specific T cells 5 mo after HCT to treat CMV, herpes simplex virus, and BK virus.

ARDS of unknown origin occurred in P16 at 3 mo after HCT and was treated with ruxolitinib for 17 mo. Sinusoidal obstruction syndrome occurred in P15 following busulfan-based conditioning. Kidney injury occurred in three patients: acute kidney injury in two (P1 and P6) and chronic kidney disease (CKD) in P14, the latter requiring ongoing hemodialysis three times weekly. Reported kidney injury was most likely transplant related, possibly in the context of virostatic treatments during CMV reactivation. Additionally, P14 experienced posterior reversible encephalopathy syndrome, although no connection with CKD was reported. Furthermore, idiopathic neuropsychiatric episodes occurred in P8 after both HCTs, requiring temporary antipsychotic medication.

Despite high morbidity of distinct patients, we cannot pinpoint a single treatment-related factor associated with OS. Health status or comorbidity scores at HCT were not assessed; however, previously reported impaired health status for P1 (9) and severe illness in P3, both of whom died after HCT, as well as a beneficial outcome in P15—transplanted preemptively—suggest an advantage of timely diagnosis and treatment, although more data are required to determine the effects of preemptive HCT.

### HCT achieves proper immune reconstitution in patients with CARMIL2 deficiency

**Figure 2.**
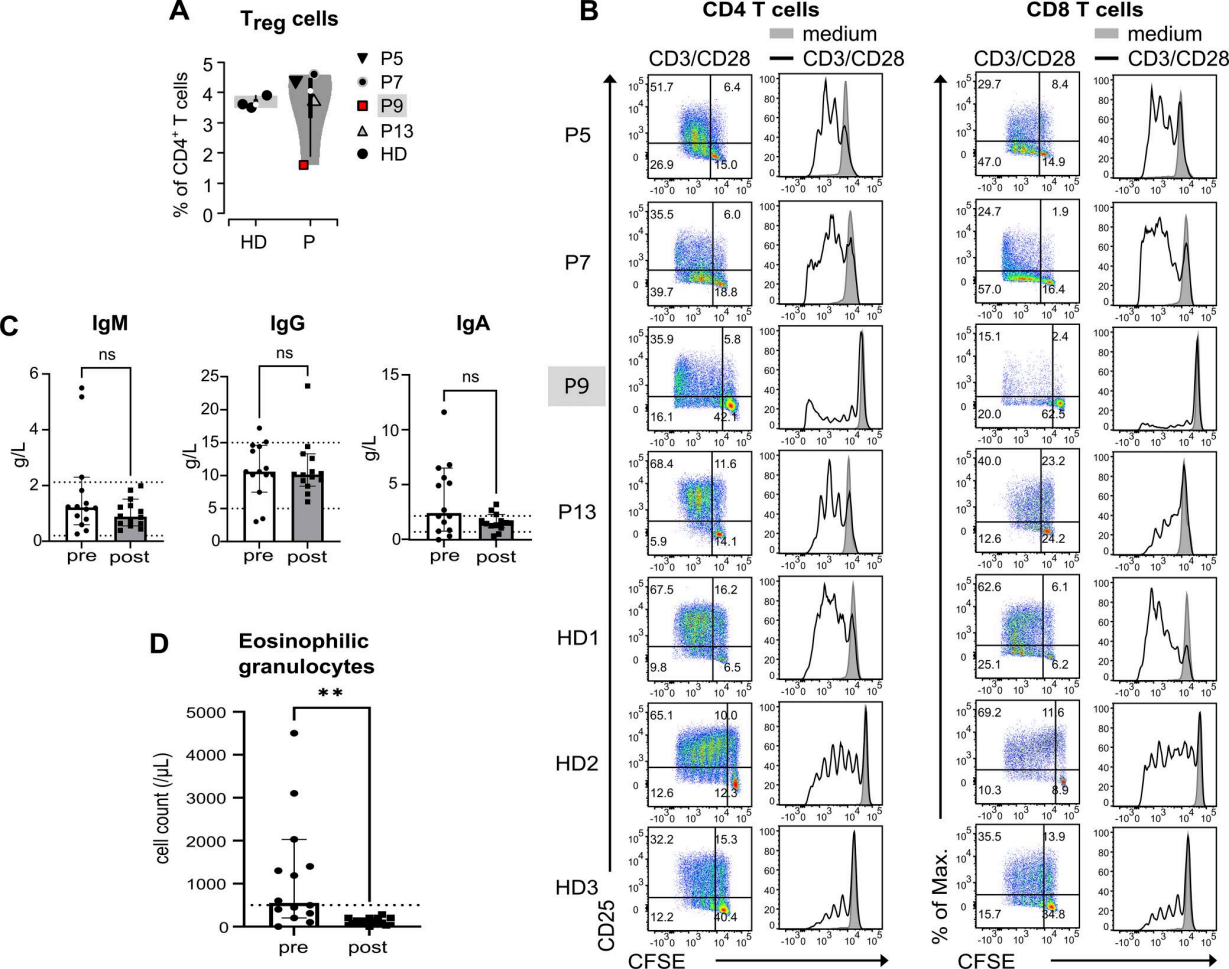
**HCT achieves proper immune reconstitution in CARMIL2 deficiency. (A and B)** Flow cytometry results show normalized CD4^+^CD25^+^CD127^low^FOXP3^+^ Treg counts (A) and CD4 and CD8 T cell proliferation for P5, P7, and P13 assessed by CD25 surface expression and CFSE dilution in medium cultured PBMC and after stimulation with anti-CD3/CD28 (CD3/CD28) for 5 days (pseudocolor plots, left panels and overlay histograms, right panels), compared to respective healthy controls (B). T cell proliferation results are detailed in Fig. S3. HD1 is a travel control for P5 and P7, and HD3 is the healthy control for P13. P9 with mixed chimerism, emphasized by a gray background, shows reduced T cell proliferation compared to HD2. **(C)** Serum immunoglobulin values for IgM, IgG, and IgA show no statistically relevant reduction after HCT using a paired, nonparametric, two-tailed statistic test (P < 0.05, ns = not significant). **(D)** Peripheral blood eosinophilic granulocyte counts are normalized in survivors after HCT, showing a significant reduction using a paired, nonparametric, two-tailed statistic test (P < 0.05). ** is defined as ** P < 0.01. HD, healthy control.

**Figure S2.**
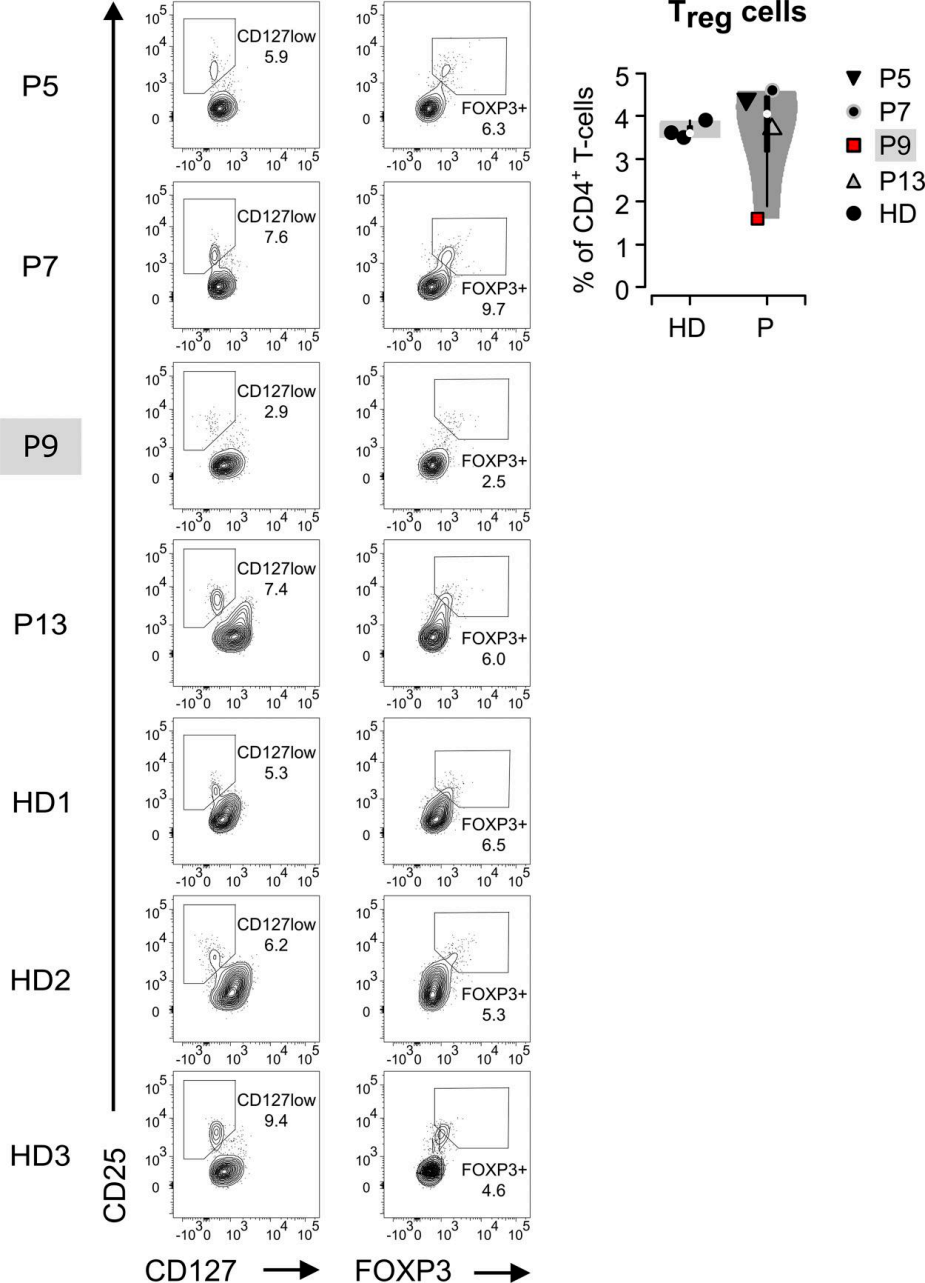
**Tregs after HCT.** Contour plots of CD25^+^CD127^low^ and CD25^+^FOXP3^+^ Treg gated on single live CD4 T cells and summary of CD4^+^CD25^+^CD127^low^FOXP3^+^ Treg percentages. Gray background depicts mixed chimerism. HD, healthy control; P patient.

**Figure S3.**
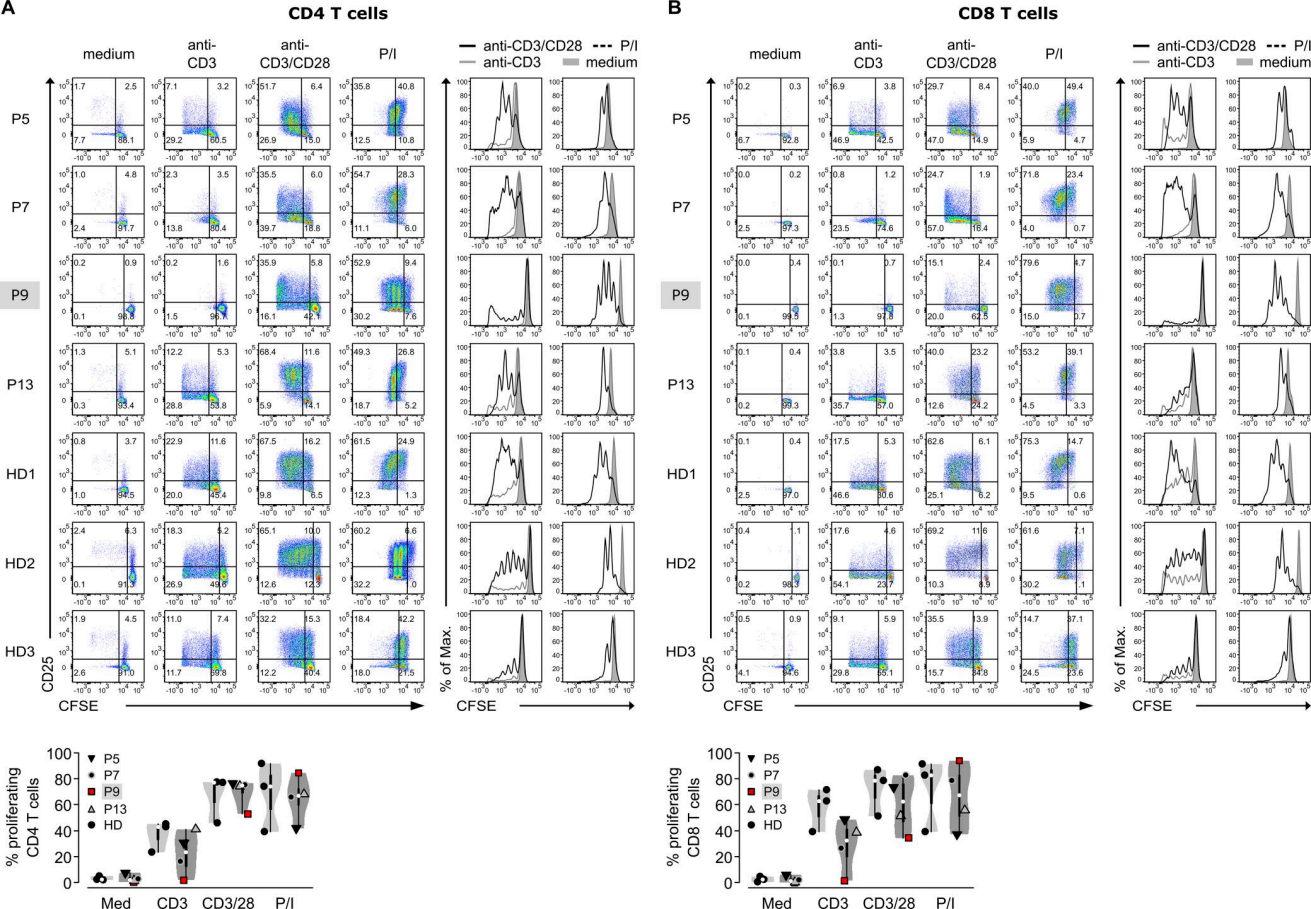
**T cell proliferation after HCT. (A and B)** Pseudocolor plots of CD25 expression and CFSE dilution and histogram of CFSE dilution on (A) CD4 and (B) CD8 T cells without (medium) and after stimulation with anti-CD3, anti-CD3/CD28 (duplicated in Fig. 2 B), or PMA/ionomycin (P/I) for 5 days and summary graphs of proliferating CD4 and CD8 T cells. Gray background depicts mixed chimerism. PMA, phorbol 12-myristate 13-acetate.

Median immunologic follow-up for surviving patients was 25 mo (range: 4–186; Table 2). No immunologic assessments were available for deceased patients. No T cell deficiency (defined as CD3 <500/μl, CD4 <250/μl, and CD8 <250/μl) was reported after HCT. Where available, counts of Tregs (Fig. 2 A and Fig. S2), CD4^+^CD45RO^+^ and CD8^+^CD45RO^+^ memory T cells, as well as T cell proliferation assays (Fig. 2 B and Fig. S3) were normal after HCT in all but P9, with mixed donor chimerism, who showed reduced Tregs and CD4^+^ memory T cells and an impaired T cell proliferation response upon CD3/CD28 co-stimulation (+13 mo; Fig. 2 B and Fig. S3).

**Table 2.** White blood cell counts pre- and post-HCT

Patient	Leukocytes (cells/μl)		Lymphocytes (cells/μl)		Neutrophils (>1,500/μl)		Eosinophils (<500/μl)		CD3 (>500/μl)		CD4 (>250/μl)		CD8 (>250/μl)		CD19 (>200/μl)		CD16CD56 (cells/μl)	
	Pre	Post	Pre	Post	Pre	Post	Pre	Post	Pre	Post	Pre	Post	Pre	Post	Pre	Post	Pre	Post
1	14,900	-	6,741	-	6,240	-	-	-	5,774	-	2,695	-	2,715	-	797	-	81	-
2	-	-	2,200	-	-	-	-	-	1,700	-	1,320	-	*140*	-	200	-	300	-
3	24,200	-	14,800	-	6,900	-	*900*	-	7,426	-	5,076	-	2,068	-	1,034	-	188	-
4	9,600	5,870 (6)	3,946	1,491 (6)	4,040	3,540 (6)	310	140 (6)	2,258	910 (6)	1,132	472 (6)	804	413 (6)	1,085	408 (6)	584	163 (6)
5	21,100	5,000 (7)	10,300	3,100 (7)	4,300	*1,100* (7)	*4,500*	200 (7)	8,106	2,625 (7)	4,532	502 (7)	2,369	1,860 (7)	2,163	337 (7)	92	131 (7)
6	10,500	6,000 (22)	3,730	1,900 (22)	5,700	3,200 (22)	100	200 (22)	2,985	1,501 (22)	1,164	465 (22)	1,379	645 (22)	391	266 (22)	34	95 (22)
7	12,700	7,400 (19)	5,600	3,200 (19)	3,100	3,600 (19)	*3,100*	100 (19)	4,793	2,656 (19)	2,660	755 (19)	1,680	1,760 (19)	761	416 (19)	39	86 (19)
8	12,140	8,070 (32)	3,070	1,710 (32)	6,150	5,460 (32)	*2,030*	280 (32)	2,163	1,232 (32)	1,674	613 (32)	499	610 (32)	654	351 (32)	96	216 (32)
9	10,200	6,500 (34)	2,700	3,690 (34)	6,900	2,180 (34)	450	200 (34)	2,052	2,448 (24)	1,350	1,530 (24)	567	646 (24)	567	884 (24)	86	48 (24)
10	12,400	8,700 (24)	7,700	4,500 (24)	3,600	3,500 (24)	*500*	200 (24)	6,082	3,867 (24)	3,895	1,866 (24)	1,873	1,793 (24)	2,010	1,157 (24)	62	64 (24)
11	14,000	2,500 (20)	7,400	1,800 (20)	5,700	*400* (20)	*600*	0 (20)	5,828	1,446 (20)	3,412	740 (20)	2,207	675 (20)	1,963	246 (20)	222	71 (20)
12	12,200	5,900 (12)	3,300	2,700 (12)	8,000	2,300 (12)	0	100 (12)	3,240	1,485 (12)	1,564	282 (12)	1,298	475 (12)	471	341 (12)	304	405 (12)
13	10,000	4,900 (47)	2,800	2,340 (47)	5,800	2,070 (47)	*1,190*	80 (47)	2,548	2,025 (23)	1,120	950 (23)	1,148	925 (23)	168	425 (23)	50	30 (23)
14	14,050	3,180 (26)	7,550	1,840 (26)	5,250	*880* (26)	200	120 (26)	4,694	1,137 (26)	2,379	558 (26)	1,958	515 (26)	758	*172* (26)	211	494 (26)
15	10,300	9,130 (40)	6,700	3,090 (40)	1,700	5,110 (40)	400	90 (40)	6,124	2,802 (40)	4,010	1,937 (40)	2,041	712 (40)	729	550 (40)	365	162 (40)
16	32,200	9,690 (70)	26,400	3,940 (70)	2,900	5,000 (70)	*1,300*	50 (70)	1,3944	2,974 (70)	9,072	1,750 (70)	4,200	1,067 (70)	2,352	484 (70)	336	339 (70)
17	8,900	3,720 (186)	5,400	2,696 (186)	1,500	*790* (186)	1,400	30 (186)	4,698	2,000 (186)	2,754	1,253 (186)	1,836	722 (186)	540	603 (186)	54	92 (186)

All cell counts are reported in cells/µl. Sampling time points in months after HCT are provided in parentheses. Values outside the provided normal range are shown in italics.

Similarly, CD19^+^ B cell deficiency (<200/μl) was reported only in P14 (+26 mo), and no patient required immunoglobulin replacement therapy (IGRT) beyond 24 mo after HCT. In contrast, 11 patients had received immunoglobulin treatment pre-HCT, even though hypogammaglobulinemia was only reported in one. Immunoglobulin results (Fig. 2 C and Table 3) show no significant differences for IgM (median pre-/post-HCT = 1.21/0.88 g/L; P = 0.2474), IgG (median = 10.6/10.2 g/L; P = 0.5830), or IgA (median = 2.24/1.40 g/L; P = 0.0574) in surviving patients compared with matched pre-HCT values. When assessed, patients showed positive serologic vaccination responses for tetanus (eight patients), diphtheria (five patients), and mumps, measles, rubella, and varicella (four patients) after HCT.

**Table 3.** Immunoglobulin values pre- and post-HCT

Patient	IgM (0.20–2.10 g/L)		IgG (5.0–15.0 g/L)		IgA (0.30–2.0 g/L)		IgE (IU/ml)	
	Pre	Post	Pre	Post	Pre	Post	Pre	Post
1	*2.40*	-	11.3	7.47 (1*)	*6.65*	-	<2.0	-
2	-	*2.59*	-	13.5	-	*11.6*	-	63
3	1.21	-	10.5	-	0.92	-	64	-
4	0.79	0.74 (5)	*17.2*	10.2 (17)	*6.28*	-	90	-
5	1.24	0.39 (7)	10.1	6.02 (7)	1.48	0.31 (7)	158	5.3 (7)
6	0.91	1.28 (22)	14.6	13.3 (22)	*1.98*	1.00 (22)	-	-
7	1.82	0.64 (19)	13.7	12.6 (19)	*4.74*	1.24 (19)	*228*	60 (19)
8	1.15	0.55 (28)	10.6	7.20 (28)	0.72	0.47 (28)	-	-
9	1.23	0.86 (34)	7.49	9.22 (34)	*1.98*	1.35 (34)	-	-
10	0.59	0.90 (24)	10.1	9.80 (24)	*2.50*	1.40 (24)	5,1	-
11	*2.30*	0.63 (22)	14.4	8.40 (22)	*5.20*	1.60 (22)	<2.0	-
12	*5.18*	1.83 (26)	15.1	10.6 (26)	*6.02*	*2.96* (26)	-	-
13	*5.50*	0.44 (47)	10.6	10.1 (47)	*10.7*	1.43 (47)	-	*406* (47)
14	1.36	1.07 (26)	11.1	9.35 (26)	4.54	1.58 (26)	0	0 (26)
15	0.26	1.22 (40)	*3.46*	10.3 (40)	0.31	1.18 (40)	-	-
16	0.38	1.51 (71)	*3.01*	14.4 (71)	*0*	*2.47* (71)	-	-
17	1.19	1.99 (186)	10.1	*23.6* (186)	0.81	*2.13* (186)	-	52.5 (186)

Sampling time points in months after HCT are provided in parentheses. Values outside the provided normal range are shown in italics.

Neutropenia (<1,500/μl) was present in four patients after HCT (Table 2) and was attributed to ethnic neutropenia of the donor for P17 (mother, no genetic verification available).

Overall, we report proper immune reconstitution in surviving patients with full donor chimerism, whereas mixed chimerism was associated with reduced T cell proliferation in response to CD28 co-stimulation and reduced memory CD4^+^ T cells in P9. Thus, HCT is curative for CARMIL2 deficiency.

### HCT corrects secondary eosinophilia and anemia in patients with CARMIL2 deficiency

Eosinophilia (≥500/μl) reported in eight patients pre-HCT (Table 2 and Table S1) was more prevalent in this cohort than expected from literature (2, 6, 12) and resolved in all cases after HCT, showing a significant reduction of median eosinophilic granulocyte counts in surviving patients from 550/μl (range: 0–4,500/μl) pre- to 110/μl (range: 0–280/μl) post-HCT (P = 0.0012; Fig. 2 D).

Anemia is mostly attributed to secondary iron deficiency due to gastrointestinal manifestations of CARMIL2 deficiency (6) and was also more prevalent in our cohort (seven patients) than expected from the literature. Hemoglobin levels were not quantified in the survey; however, no autoantibodies were reported, and BM sampling, where available, showed normal hematopoiesis. After HCT, four patients had persistent anemia, including P11 with persisting poor graft function, reportedly compatible with secondary myelodysplastic syndrome. P9 with mixed donor chimerism had persistent anemia due to a preexisting hematologic condition (β-thalassemia minor; no erythrocyte chimerism available), and P14 had a renal anemia due to CKD receiving regular treatment with erythropoietin.

Overall, HCT can correct secondary hematopoietic features such as eosinophilia and anemia in CARMIL2 deficiency.

### HCT improves severe infection susceptibility in patients with CARMIL2 deficiency

All reported infections are provided in Table S1. Pathological infection susceptibility was reported in nine patients pre-HCT and improved in all except P11 with limbic encephalitis. Respiratory tract infections continued to be the most prevalent after HCT, including *Pneumocystis jirovecii* pneumonia (PjP; P1) during multifactorial respiratory failure leading to death, whereas PjP was reported in three patients pre-HCT. P8, following a 2-year treatment for *Mycobacterium avium* complex pneumonia, requires ongoing prophylaxis with cotrimoxazole and penicillin. Mycobacterial infections also occurred in three patients pre-HCT, and no reactivation was reported after HCT, including P13, who received intensified immunosuppression for aGVHD. P14 with renal-replacement therapy for CKD received penicillin prophylaxis for 2 years after HCT. SARS-CoV-2 infections were reported for P4 (12 mo) and P10 (13 mo) with mild disease. Viral skin infections (warts: three patients, molluscum: one patient) resolved in all patients after HCT.

In summary, infections observed after HCT are largely procedure related and HCT can improve pathological infection susceptibility in CARMIL2 deficiency.

### HCT corrects inflammatory, autoimmune, and allergic manifestations associated with CARMIL2 deficiency

Skin disease was present in 10 out of the 14 surviving patients pre-HCT, including atopic dermatitis (six patients), psoriasis-like lesions (three patients), and orofacial granulomatosis, seborrheic, or pruritic dermatitis (each one patient), which substantially improved or resolved after HCT (Table S1). Pre-HCT gastrointestinal tract manifestations were reported in nine surviving patients, with resolution in all cases after HCT (Table S1). P4 had severe IBD pre-HCT requiring treatment with adalimumab, which resolved after HCT with no further treatments. Inflammatory airway disease significantly improved in two patients (P5 and P7) and no allergies have been reported in the entire cohort after HCT. Asymptomatic autoantibodies against thyroid and pancreatic β cells (not further specified) were reported for P5 and P7 pre-HCT and resolved after HCT, potentially representing false-positive serology results under IGRT (25).

In conclusion, HCT corrects inflammatory, autoimmune, and allergic manifestations of CARMIL2 deficiency following immune reconstitution.

### HCT halts progression and leads to regression of otherwise treatment-refractory EBV+ SMTs

EBV+ SMTs represent rare manifestations of ectopic EBV infections in immunodeficient hosts, exhibit histological distinctions from other types of SMTs, and are largely treatment-refractory (26, 27). They are typically observed in patients with secondary immunodeficiency, notably due to HIV infection, but are also observed in patients with IEIs affecting T and NK cell immunity (16, 26), including 17% of patients with CARMIL2 deficiency (5).

Pre-HCT screening for EBV+ SMTs occurred in seven patients by MRI (five patients), CT (four patients), ultrasound (four patients), PET-CT (two patients), and endoscopy (one patient). EBV+ SMTs were present in five patients pre-HCT (Table 4), and all received grafts from EBV seropositive donors (IgM^−^ and IgG^+^). After HCT, one patient achieved complete remission (P13, 20%), two showed a partial remission (P5 and P7, 40%), one had stable disease (P8, 20%), and one suffered from progressive disease (P1, 20%), contributing to an early multifactorial respiratory failure and subsequent death 1 mo after HCT. This patient had shown extensive disease prior to HCT, affecting the brain, lungs, large intestine, liver, gall bladder, kidney, and spine (L5/S1).

**Table 4.** Overview of patients with EBV+ SMTs and outcome after HCT

Patient	Localization	Pre-HCT		Post-HCT			Donor EBV status
		Treatment	Outcome	Latest follow-up (months after HCT)	EBV+ SMTs present?	Outcome	
1	Brain, lungs, large intestine, liver, gall bladder, kidney, and L5/S1 spinal lesion	None	-	Deceased	Yes	Progression of lung SMTs	IgM−IgG +
5	Bilateral adrenal glands	None	-	21	Yes	Partial response	IgM−IgG +
7	Bilateral adrenal glands	None	-	28	Yes	Partial response	IgM−IgG +
8	Appendix	None	Stable disease	32	Yes	Appendectomy 9 mo after 2nd HCT Complete remission	IgM−IgG +
13	Lung, gastrointestinal tract	Endoscopic removal	Relapse	24	No	Complete remission without further treatment	IgM−IgG +

**Figure 3.**
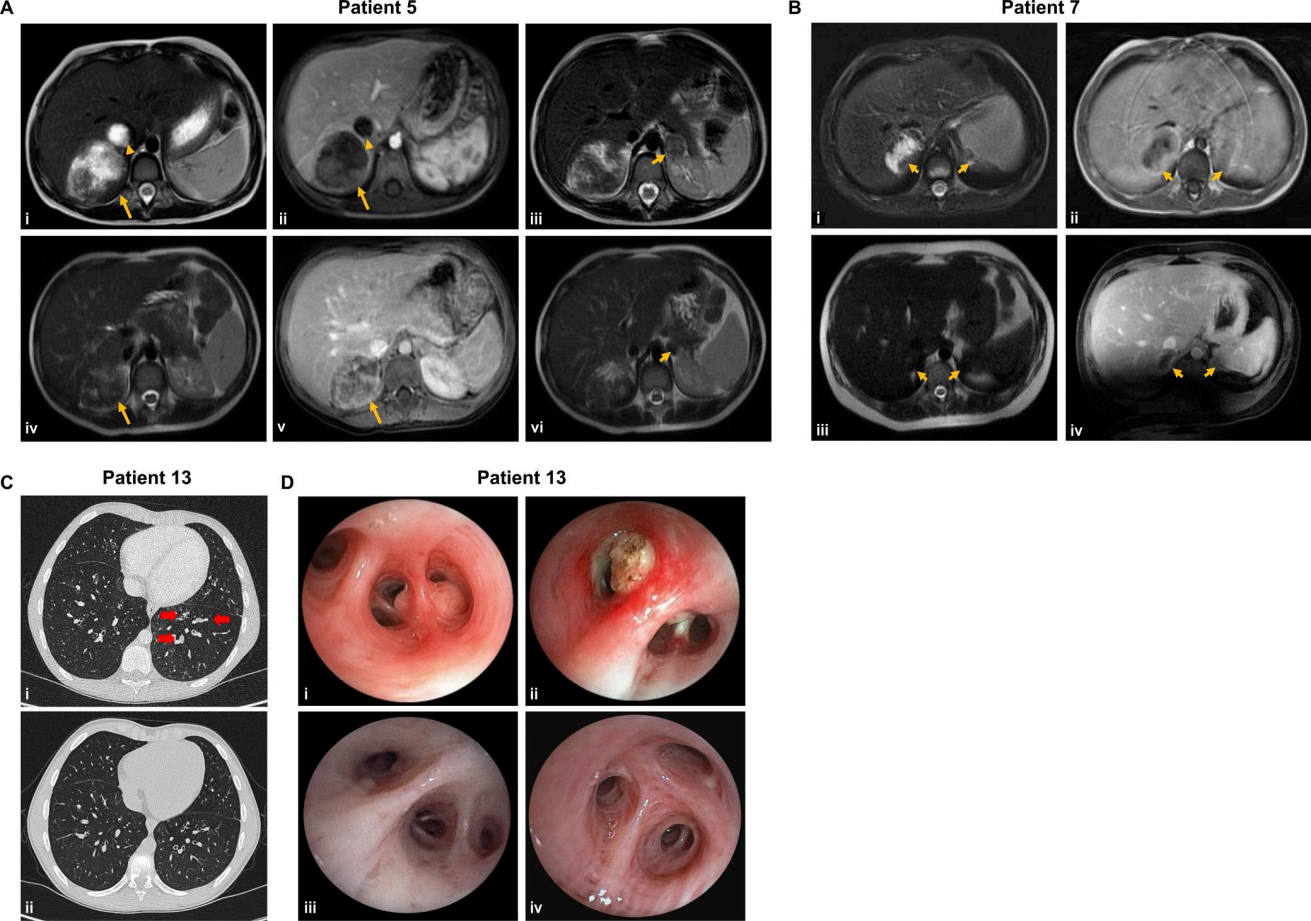
**HCT halts progression and leads to regression of otherwise treatment-refractory EBV+ SMTs. (A)** MRI images pre- (i, ii, iii; upper row) and 21 mo post-HCT (iv, v, vi; lower row) for P5: T2 weighted axial (i) and T1 weighted post-contrast axial (ii) images demonstrate two lesions in right adrenal gland (larger 62 mm) with T2 heterogenous signal and peripheral contrast enhancement (arrows and arrowhead), the smaller compressing the inferior vena cava (VCI). T2 weighted axial (iii) image shows lesion in left adrenal gland (arrow) with similar signal changes. After HCT, T2 weighted axial (iv) and T1 weighted post-contrast axial (v) images show a decrease in size of the larger right adrenal lesion (57 mm) and disappearance of the VCI-compressing lesion. T2 weighted axial image (vi) shows a similar decrease in size on the left adrenal lesion (size not reported). **(B)** MRI images pre- (i, ii; upper row) and 28 mo post-HCT (iii, iv; lower row) for P7: T2 weighted axial (i) and T1 weighted post-contrast axial (ii) images show bilateral adrenal lesions (right: 38 × 32 × 38 mm, left: size not reported) with T2 heterogenous signal and peripheral contrast enhancement (arrows). Post-HCT T2 weighted axial (iii) and T1 weighted post-contrast axial (iv) images show decrease in size of bilateral adrenal lesions (right: 25 × 17 mm; left: size not reported). **(C)** Axial CT images for P13 show multiple bronchial obstructions by intraluminal leiomyomas pre-HCT (i; red arrows) with complete resolution 10 mo after HCT (ii). **(D)** Bronchoscopy images for P13 show multiple intraluminal bronchial leiomyomas 2 mo pre-HCT (i), which relapsed following removal noted 1 mo pre-HCT (ii). Subsequent complete remission was seen at 11 mo (iii) and 24 mo (iv) after HCT. Images are representative and may not depict the same airway localization for pre- and post-HCT findings.

P5 and P7 had EBV+ SMTs in bilateral adrenal glands, which showed partial response to HCT on latest available radiographic follow-up performed at 21 and 28 mo, respectively (Fig. 3, A and B). P13 showed relapsing EBV+ SMTs after endoscopic removal of pulmonary and intestinal masses pre-HCT, and complete remission was apparent on bronchoscopy and CT imaging 24 mo after HCT without receiving further treatment and despite intensified immunosuppression for aGVHD (Fig. 3, C and D). P8 was diagnosed with EBV+ SMT as an incidental finding during appendectomy for persistent abdominal discomfort following a second HCT, which were retrospectively also identified on abdominal imaging studies conducted before HCT. At last follow-up, complete remission was noted 32 mo after surgical removal (images not available).

We observed no discernible impact of conditioning regimens on EBV+ SMT outcomes, although P13 with complete remission received no lymphodepletion by serotherapy. Immune reconstitution did not differ between patients with and without EBV+ SMTs after HCT, and we observed neither relevant EBV viremias nor other EBV-related complications such as post-transplant lymphoproliferative disease.

In summary, our data indicate that HCT brings a clinical benefit in CARMIL2-deficient patients with refractory EBV+ SMTs.

## Discussion

We report the first international cohort of CARMIL2-deficient patients treated with allogeneic HCT, demonstrating good OS, successful primary immune reconstitution, and significant clinical benefit. We observed the resolution of secondary eosinophilia and anemia, improvement of pathological infection susceptibility, and correction of skin and intestinal inflammatory, autoimmune, and allergic manifestations. Importantly, HCT-induced regression of otherwise treatment-refractory EBV+ SMTs in three out of five patients. While considering the burden of complications and mortality in CARMIL2-deficient patients, our data indicate that HCT should be offered in specialized centers for these patients upon diagnosis (5). The use of immune deficiency and dysregulation activity score or comorbidity scores may further support clinicians in their assessment regarding HCT indication (28, 29, 30). Given the overall cohort size, heterogeneity of HCT modalities and the early post-HCT mortality of three patients, a systematic comparison of distinct conditioning regimens is beyond the scope of this report, and no causality for observed tendencies can be deduced. In any case, we suggest performing therapeutic drug monitoring for busulfan and to include serotherapy into the conditioning regimen (31, 32).

Skin disease is considered a hallmark of CARMIL2 deficiency (5) with recurrent skin infections, including abscesses, warts, and signs of allergic skin inflammation such as eczematous dermatitis (4) or rarely photodermatitis (10). The association between CARMIL2 deficiency and skin disease is further emphasized by the initial report describing downregulation CARMIL2 in psoriasis vulgaris (1) and a possible role of CARMIL2 in wound healing (33). However, we here report excellent resolution of immunodeficiency and allergic inflammation of the skin, as well as the intestine after HCT. This observation suggests that most patients’ clinical barrier phenotypes, predominantly of the gut and skin, are driven by impaired immune cell functions and not by impaired epithelial tissue intrinsic functions, emphasized by the resolution of inflammatory manifestations in P5, P7, and P13 following immune reconstitution, including Tregs. In addition, and to reduce the risk of skin GVHD in CARMIL2 deficiency, we suggest treating eligible patients with dupilumab pre-HCT, which in our hands shows good efficacy (unpublished data), although one patient was reported with paradoxical psoriasis post-dupilumab treatment, prompting treatment discontinuation (unpublished data) (34).

Our data provide strong evidence for HCT as a therapeutic option for EBV+ SMTs not only in CARMIL2 deficiency but in IEI as such. We show clear improvement in two and complete remission in one patient following immune reconstitution with full donor chimerism. Similarly, resolution of multifocal EBV+ SMTs was reported in one patient with GATA2 deficiency after HCT (35). Close monitoring of EBV in patients with an increased susceptibility has been recommended especially during HCT and may be helpful in identifying early manifestations of lymphoproliferative disorders or malignancy (36, 37). Importantly, and at least in this cohort of CARMIL2-deficient patients with EBV+ SMTs, we did not observe unusual EBV reactivation or EBV-related lymphoproliferative complications during HCT. We thus recommend screening all patients with CARMIL2 deficiency for EBV+ SMTs, as this otherwise treatment-refractory manifestation is a strong indication for therapeutic HCT. Furthermore, we emphasize the necessity for genetic and immunological work up in all patients with atypical presentation of viral infections such as EBV+ SMTs (4, 16, 38).

Eosinophilia is frequently observed in patients with primary atopic disorders such as CARMIL2 deficiency as a sign of a dysregulated T helper type 2 immunity, although tissue and peripheral blood eosinophilic granulocyte counts may not correlate with disease severity (39). The cytoskeleton is paramount in executing successful immune responses, and an increasing number of IEIs are being linked to actin-related defects (40). Interestingly, actin dysregulation and peripheral eosinophilia show a strong correlation as reviewed by Kim et al. (41) who compared Wiskott-Aldrich syndrome (WAS), DOCK8 deficiency, and CARMIL2 deficiency. Therefore, eosinophilia should be recognized as a potential sign of IEIs and primary atopic disorders. Eosinophilia resolved after HCT in CARMIL2-deficient patients, as similarly reported for DOCK8 deficiency (42).

Global and lineage-specific donor chimerisms are commonly used as a marker for HCT success, although functional assays are required to prove sufficient immunologic correction (22). An in-depth review of the immunological phenotype after HCT is beyond the scope of this report given differences in follow-up practices among reporting institutions. Thus, further immunological analysis and post-HCT dynamics of immune reconstitution are needed. Still, our data indicate proper immune reconstitution in patients exhibiting full donor chimerism, whereas P9, with mixed chimerism, shows reduced counts of Treg and CD4^+^ memory T cells and an impaired T cell proliferation upon CD28 co-stimulation after HCT. Nevertheless, this patient requires neither prophylactic treatments nor immunoglobulin substitution, and all symptoms such as pruritic dermatitis, IBD, pathological infection susceptibility, and failure to thrive have substantially improved after HCT. Therefore, we see no clear detrimental effect of mixed chimerism in CARMIL2 deficiency. However, further data are required to assess long-term outcomes of mixed chimerism or lineage-specific chimerism, particularly given that P9 and P10 show a consistent decrease of donor chimerism over time, possibly with long-term consequences. Long-term outcomes for lineage-specific chimerism for IEI have been reported in select diseases, showing a benefit of full donor chimerism for WAS (43). Akin to that, the authors suggest to aim for full donor chimerism in HCT in DOCK8 deficiency, despite lacking clear evidence for an unfavorable effect of mixed chimerism (42). Our data suggest that mixed chimerism may be tolerated in patients with preexisting organ damage necessitating reduced-toxicity conditioning regimens.

In conclusion, allogeneic HCT substantially addresses CARMIL2 immunodeficiency, immune dysregulation, and EBV+ SMTs and is a feasible up-front therapeutic option for this severe IEI, which should be offered preemptively given the high morbidity and mortality of the disease.

## Materials and methods

### Data acquisition

The study was conducted in accordance with the Declaration of Helsinki. Data were acquired through a retrospective online survey (LimeSurvey GmbH) located on a secured server at the LMU Munich, Germany. A copy of the survey is available upon reasonable request. Physicians were invited to submit patients from previous publications (5) or personal contacts. All patients with genetically confirmed CARMIL2 deficiency receiving HCT for any indication were included irrespective of follow-up time. Patients with known additional genetic disorders were excluded. No personal data except gender and *CARMIL2* variants were collected or reported in accordance with the study protocol. Primary objectives were the detailed description of modalities, morbidity, mortality, and outcome of HCT for CARMIL2 deficiency. The study was approved by the ethics committee at the LMU Munich, Germany (Project #22-0840). Acute or chronic GVHD and graft failure were assessed according to the current EBMT criteria (24). Engraftment was defined for cell counts of leukocytes (>1,000/μl), neutrophils (>500/μl), and platelets (>50,000/μl) on 3 consecutive days. Patients were censored at latest available follow-up. Individual patients consented to Institutional Review Board protocols or patient’s or caregiver’s informed consent was obtained for the use of radiographic images or prospective data at their respective sites.

### Isolation and stimulation of peripheral blood mononuclear cells (PBMCs)

PBMCs were isolated from heparinized blood samples by Ficoll-Paque (Cytiva) density centrifugation and maintained in RPMI1640 supplemented with 2 mM GlutaMAX, 100 U/ml penicillin, 100 µg/ml streptomycin, and 10% FCS (Thermo Fisher Scientific) at 37°C and 5% CO_2_. For T cell proliferation, PBMCs were labeled with 2.5 µM carboxyfluorescein succinimidyl ester (CFSE, Thermo Fisher Scientific) and stimulated with anti-CD3–coupled beads (anti-Biotin MACSiBeads, Miltenyi Biotec, coupled with Biotin-anti-CD3, OKT3) at a ratio of 5:1 with and without 1 µg/ml anti-CD28 antibody (CD28.2, both Thermo Fisher Scientific) or with 0.5 ng/ml phorbol 12-myristate 13-acetate and 1 µM ionomycin (Sigma-Aldrich) for 5 days in a round-bottom 96-well plate.

### Flow cytometry

Tregs were stained with APC-H7-anti-CD3 (SK7, 1:50), APC-anti-CD4 (SK3, 1:100), PE-anti-CD25 (M-A251, 1:50) (all Becton Dickinson [BD]), and FITC-anti-CD127 (eBioRDR5, 1:50, Thermo Fisher Scientific). Intracellular staining with PerCP-Cy5.5-anti-FOXP3 (PCH101, 1:25) was performed using FOXP3 staining kit (Thermo Fisher Scientific). T cell proliferation was measured by CFSE dilution of stimulated PBMCs with APC-H7-anti-CD3 (SK7, 1:50), APC-anti-CD4 (SK3, 1:50), PacB-anti-CD8 (RPA-T8, 1:50), and PE-anti-CD25 (M-A251, 1:25, all BD) 5 days after stimulation as described previously (42). For analysis of CARMIL2 expression, PBMCs were stained with APC-H7-anti-CD3 (SK7, 1:50), APC-anti-CD4 (SK3, 1:50), PacB-anti-CD8 (RPA-T8, 1:50) (all BD), and PC7-anti-CD19 (J3-119, 1:100, Beckman Coulter). Intracellular staining was done with PE-anti-CARMIL2 (EM53, 1:10, EXBIO) or isotype control PE-anti-mouse IgG1 (MOPC-21, 1:10, BD) using Cytofix/Cytoperm buffer set from BD. Data were acquired on a BD FACSCanto II flow cytometer. Data analysis was performed with FlowJo software (TreeStar).

### Statistical analysis

Kaplan–Meier survival curve and statistical analyses were performed with Prism Version 10 (GraphPad Software, LLC; personal license) or with the software environment R (version 4.4.0) using a paired, nonparametric, two-tailed Wilcoxon test or an unpaired, nonparametric, two-tailed Mann–Whitney test accepting P < 0.05 as significant. Central tendencies are reported as median values.

### Online supplemental material

The supplementary material includes detailed descriptions of the clinical phenotypes of CARMIL2-deficient patients (Table S1) and their treatments before and after HCT (Table S2), lineage-specific chimerism results for CD3 T and CD19 B cells (Fig. S1), and extended immunological studies of Tregs (Fig. S2) and T cell proliferation (Fig. S3) in selected patients following HCT.

## Supplementary Material

Table S1 shows an overview of symptoms.

Table S2 shows an overview of treatments.

## Data Availability

Deidentified data or a copy of the online survey may be available upon reasonable request by email to the corresponding author.
