# Supplementary material for: Allogeneic hematopoietic cell transplantation is curative in CARMIL2 deficiency
Source: J Hum Immun. 2026 Jul 29;2(5):e20250200. doi: 10.70962/jhi.20250200 (PMC13418249; doi:10.70962/jhi.20250200)
Supplement: Table S1 — shows an overview of symptoms. [file jhi_20250200_tables1.docx]

**Table S1: Overview of symptoms**

|  | **Hematology** | | **Skin disease** | | **GI tract** | | **Infections** | | **Other** | |
| --- | --- | --- | --- | --- | --- | --- | --- | --- | --- | --- |
| **Patient** | Pre | Post | Pre | Post | Pre | Post | Pre | Post | Pre | Post |
| **1** | Anemia, eosinophilia | None | None | NA | IBD  (CD; endoscopy performed, NA) | NA | LRTI: S. marcescens, K. pneumoniae, E. coli (ET tube) GI: Cryptosporidium CNS: Dissiminated M.chelonae infection CMV viremia, C. albicans fungemia | LRTI: PjP, E. faecium pneumonia; CMV | Failure to thrive,  Panhypopituitarism, brain mass (subclinical seizures, NIII palsy Pulmo: recurrent interstitial pneumonitis; mechanical ventilation | AKI (Grade 3);  progression of EBV+ SMTs in lungs; multifactorial respiratory failure,  **Deceased** |
| **2** | None | NA | Atopic dermatitis, psoriasis-like lesions | NA | IBD  (UC, endoscopy performed, NA) | NA | URTI: Rhinovius LRTI: M. tuberculosis, NTM (MAC), S. angiosus Skin: HPV | CNS: JC virus encephalitis | Retinitis, Failure to thrive Food allergies | **Deceased** |
| **3** | Anemia, eosinophilia | 1st: primary graft failure | Atopic dermatitis, psoriasis-like lesions | NA | IBD  (endoscopy: macroscopic slightly thickened esophogeal mucosa with lymphocytic and increased apoptotic phenomena; eosinophilic infiltration in colon) | NA | Pulmo: CMV (BAL) GI: Norovirus, CMV, EBV | S. aureus bacteremia, CMV, ADV, BKV | Failure to thrive | 1st: primary graft failure 2nd: ARDS d+7,  **Deceased** |
| **4** | None | None | Atopic dermatitis | None | Severe IBD  (CD; endoscopy: patchy, discontinuous ileocolonic inflammation with erosive/aphthous lesions and suspected granulomas) | None | URTI: recurrent  Skin: S. aureus infection  GI : recurrent gastroenteritis | HCT : EBV, ADV  LRTI : RSV + coronavirus NL64+OC43 (2 months); SARS-CoV-2 (12 months), | Failure to thrive Grade IIIA anaphylactic reaction to liposomal amphotericin B | Resolved failure to thrive No allergies |
| **5** | Eosinophilia | None | Psoriasis-like lesions | aGVHD, resolved | None | None | LRTI | HCT: CMV | asymptomatic thyroid and DM-I autoantibodies | No susceptibility to infections; autoantibodies not detectable |
| **6** | Anemia | None | Warts | aGVHD, resolved | IBD  (endoscopy: mild esophageal erosions and furrowing, mild gastritis) | None | LRTI: PjP, CMV | HCT: CMV, EBV | GI: chronic diarrhea | AKI; Resolved GI symptoms; Improved susceptibility to infections |
| **7** | Eosinophilia | None | Psoriasis-like lesions | aGVHD, resolved | Bloody diarrhea with eosinophilic enteropathy | None | LRTI | None | asymptomatic thyroid and DM-I autoantibodies | No susceptibility to infections;  Autoantibodies not detectable |
| **8** | Anemia, eosinophilia | None | Atopic dermatitis, severe HPV warts | None | None | GI symptoms resolved after appendectomy (EBV+ SMT) | URTIs, LRTI: H. influenza, P. aeruginosa, NTM (MAC) Skin: HPV, VZV | URTI: ongoing sinusitis, Rhinovirus LRTI: pulmonary NTM (M. chimera) post-2nd HCT, Ciprofloxacin-resistant P. aeruginosa HCT: FUO, oral candidosis, EBV | Failure to thrive, lymphadenopathy (cervical, hilar, mesenterial), Allergies (medication) nasal polyposis, chronic sinusitis Pulmo: spontaneous pneumothoraces, bronchiectasis with bronchoceles | 1st: Idiopathic psychiatric episode (paranoia, delusions); no pathogens identified, no abnormal scans.  2nd: Improved failure to thrive; Improved susceptibility to infections, improved chronic sinusitis; Osteoarthritis of hips |
| **9** | Anemia, eosinophilia | Anemia (9.2 g/dL; b-thalassemia minor) | Pruritic dermatitis, onychomycosis | Improved dermatitis, aGVHD resolved | IBD  (endoscopy: enteritis in bulbus duodeni and colon, CMV+EBV+) | None | UTRIs; LRTI: PjP GI; Skin: S. aureus | HCT: CMV  UTRI: Moraxella | Failure to thrive | Initial complications due to lung and GI disease Improved failure to thrive Improved susceptibility to infections |
| **10** | Eosinophilia | None | Atopic dermatitis | aGVHD, resolved | IBD  (CD; endoscopy: large plaque at bulbus duodeni, practically stenotic duodenum) | None | URTIs,  LRTIs: ADV, RSV, EBV | HCT: S. aureus bacteremia, CMV, ADV, MPV  URTI: Tonsillitis (21 months) LRTI: mild COVID-19 (13 months) Unknown viral infection (24 months) | Allergy: pollen | Improved susceptibility to infections |
| **11** | Eosinophilia | Pancytopenia  (compatible with secondary MDS) | Atopic dermatitis, orofacial granulomatosis | Improved | IBD  (early-onset CD; endoscopy: large ulcerative lesion in bulbus duodeni with stenotic duodenum, no pathological findings in colon) | None | URTIs, LRTIS, | LRTI: P. aeruginosa (ESBL) colonization CNS: HHV6 limbic encephalitis (viremia + spinal) HCT: CMV, HSV (viremia) | Failure to thrive, Cheilitis | persistent susceptibility to infections,  treatment-refractory epilepsy,  frequent vomiting, esophageal strictures,  failure to thrive |
| **12** | Anemia | None | Warts,  seborrheic dermatitis | None | CMV colitis, diarrhea  (endoscopy NA) | None | LRTI: CMV  Skin: HPV | LRTI HCT: CMV, EBV | None | Improved susceptibility to infections |
| **13** | Anemia, eosinophilia | mild Anemia (Hb 12.4 g/dL) | Atopic dermatitis, psoriasis-like lesions | Improved dermatitis, aGVHD resolved | chronic diarrhea  (endoscopy: gastroesophageal reflux, sooresophagitis, stenosis of duodenum, CMV+ sigmoiditis);  EBV+ SMT | aGVHD resolved,  no GI symptoms | URTIs, LRTIs: PjP, S. pyogenes, Moraxella, M. pneumoniae, NTM (M. jordanae) GI: CMV Skin: S. aureus chronic CMV & EBV | URTI & LRTI due to bronchiectasis HCT: CMV, EBV | Failure to thrive cervical lymphadenopathy, dilated bulbus aortae, aneurysm left ACA, liver cyst Bronchiectasis | HCT: engraftment syndrome; Improved failure to thrive; indirect hyper-bilirubinemia |
| **14** | Anemia | Renal anemia | Molluscum contagiosum | None | None | None | URTI: CMV, LRTI Skin: Molluscum | HCT: FUO, CMV + HSV + BKV | O2 supplementation (2-5L/min) | Improved susceptibility to infections, CKD (5, chronic dialysis), PRES,  No O2 supplementation,  Hypergonadotropic hypogonadism |
| **15** | None | None | None | None | None | None | None | GI: enterovirus HCT: FUO, EBV | None; transplanted pre-emptively | SOS  Mucositis Grade 4 |
| **16** | Anemia, eosinophilia | None | Atopic dermatitis | None | None | None | CMV, EBV (viremia) | HCT: K. pneumoniae bacteremia, CMV, EBV | Failure to thrive | Improved failure to thrive,  ARDS of unknown origin |
| **17** | Eosinophilia | None | None | None | Eosinophilic esophagitis and colitis | None | URTI, LRTI  Skin  HSV + CMV | No significant infections | Failure to thrive  Microcephaly | Improved failure to thrive |

*ADV* adenovirus*, aGVHD* acute graft-vs-host disease, *AKI* acute kidney injury, *BAL* bronchoalveolar lavage, *BKV* BK virus*, CD* Crohn’s disease, *CMV* cytomegalovirus, *CNS* central nervous system*EBV* Epstein-Barr virus, *EBV+ SMT* Epstein-Barr virus-positive smooth muscle tumors, *ECP* extracorporeal photopheresis, *FUO* fever of unknown origin, *GI* gastrointestinal tract, *HCT* hematopoietic cell transplantation (infections occurring as an acute complication), *HPV* human papilloma virus, *IBD* inflammatory bowel disease, *LRTI* lower respiratory tract infection, *MPV* metapneumovirus, *NTM* Nontuberculous mycobacteria, *PjP* pneumocystis jirovecii pneumonia, *RSV* respiratory syncytial virus, *UC* ulcerative colitis, *URTI* upper respiratory tract infection, *VZV* varicella zoster virus.
