# Supplementary material for: Allogeneic hematopoietic cell transplantation is curative in CARMIL2 deficiency
Source: J Hum Immun. 2026 Jul 29;2(5):e20250200. doi: 10.70962/jhi.20250200 (PMC13418249; doi:10.70962/jhi.20250200)
Supplement: Table S2 — shows an overview of treatments. [file jhi_20250200_tables2.docx]

**Table S2: Overview of treatments**

|  | **HCT dates** | **Immunoglobulin substitution** | | **Treatments** | |
| --- | --- | --- | --- | --- | --- |
| **Patient** | (MM/YYYY) | Pre | Post | Pre | Post |
| **1** | 02 / 2020 | No | - | M. chelonae: Linezolid, clarithromycin, amikacin CMV: Ganciclovir C. albicans: Micafungin IBD: Corticosteroids, sirolimus, infliximab | NA |
| **2** | 09 / 2018 | Yes | - | Clindamycin | NA |
| **3** | 12 / 2015 | IVIG | - | Cotrimoxazol | NA |
| **4** | 11 / 2022 | SCIg | Not after  d+100 | IBD: Adalimumab | Cotrimoxazol, aciclovir during first year |
| **5** | 08 / 2021 | IVIG | Not after  d+100 | None | On-demand bronchodilator |
| **6** | 02 / 2021 | IVIG | IVIG  (d+300) | Cotrimoxazol, aciclovir, foscarnet | Cotrimoxazol, aciclovir, letermovir during first year  aGvHD Skin: Systemic steroids, CSA + ECP (10 months) |
| **7** | 01 / 2021 | IVIG | Not after  d+100 | None | On-demand bronchodilator |
| **8** | 02 / 2022 | No | Yes  (24 months) | multiple antibiotic treatment courses no prophylaxis | Cotrimoxazol, penicillin V ongoing;  aciclovir during first two years;  Two year antibiotic course for pulmonary MAC infection |
| **9** | 10 / 2020 | IVIG | Not after  d+100 | Cotrimoxazol | NA |
| **10** | 03 / 2020 | IVIG | Not after  d+100 | Azithromycin EBV: Rituximab IBD: Prednisolon, azathioprin Iron substitution Inhalative: Budesonide | None |
| **11** | 07 / 2020 | IVIG | Not after  d+100 | Azithromycin, aciclovir EBV: Rituximab IBD: Infliximab, ustekinumab | Azithromycin (ongoing),  multiple antbiotic treatments,  extensive anticonvulsive treatment |
| **12** | 12 / 2019 | No | Not after  d+180 | Cotrimoxazol Corticosteroids CMV pneumonitis: Ganciclovir | Cotrimoxazol, aciclovir during first year |
| **13** | 09 / 2019 | No | IVIG  (d+104) | Cotrimoxazol, azithromycin,  ganciclovir, amphotericin B IBD: Prednisolon, budesonide | Intensified immunosuppression:  Cy, TAC MMF, corticosteroids, ruxolitinib (end d+528) |
| **14** | 06 / 2019 | IVIG | Not after  d+100 | Cotrimoxazol, aciclovir methylprednisolone | Multivirus-specific T cells (5 months)  Antimycotic + virostatic prophylaxis (3 months),  penicillin for 2 years,  erythropoietin for renal anemia |
| **15** | 12 / 2018 | No | Yes  (14 months) | Cotrimoxazol | None |
| **16** | 02 / 2016 | No | Yes  (23 months) | Cotrimoxazol topic diprisone | Ruxolitinib (17 months),  rituximab (1x, EBV),  ganciclovir (1x, CMV) |
| **17** | 06 / 2007 | IVIG | IVIG first year | None | None |

*aGVHD* acute graft-versus-host disease, *CMV* cytomegalovirus, *CSA* cyclosporine*, Cy* cyclophosphamide, *EBV* Epstein-Barr virus, *ECP* extracorporeal photopheresis, *IBD* inflammatory bowel disease, *IVIG* intravenous immunoglobulin substitution, *MAC* Mycobacterium avium complex, *MMF* mycophenolate mofetil, *NA* not available, *SCIg* subcutaneous immunoglobulin substitution, *TAC* tacrolimus
